# Preparation of macroporous zirconia monoliths from ionic precursors via an epoxide-mediated sol-gel process accompanied by phase separation

**DOI:** 10.1088/1468-6996/16/2/025003

**Published:** 2015-03-23

**Authors:** Xingzhong Guo, Jie Song, Yixiu Lvlin, Kazuki Nakanishi, Kazuyoshi Kanamori, Hui Yang

**Affiliations:** 1 School of Materials Science and Engineering, Zhejiang University, Hangzhou, 310027, People’s Republic of China; 2 Department of Chemistry, Graduate School of Science, Kyoto University, Kitashirakawa, Sakyo-ku, Kyoto 606-8502, Japan

**Keywords:** porous materials, zirconia monoliths, sol-gel, phase separation, heat-treatment, solvothermal treatment

## Abstract

Monolithic macroporous zirconia (ZrO_2_) derived from ionic precursors has been successfully fabricated via the epoxide-mediated sol-gel route accompanied by phase separation in the presence of propylene oxide (PO) and poly(ethylene oxide) (PEO). The addition of PO used as an acid scavenger mediates the gelation, whereas PEO enhances the polymerization-induced phase separation. The appropriate choice of the starting compositions allows the production of a macroporous zirconia monolith with a porosity of 52.9% and a Brunauer–Emmett–Teller (BET) surface area of 171.9 m^2^ · g^−1^. The resultant dried gel is amorphous, whereas tetragonal ZrO_2_ and monoclinic ZrO_2_ are precipitated at 400 and 600 °C, respectively, without spoiling the macroporous morphology. After solvothermal treatment with an ethanol solution of ammonia, tetragonal ZrO_2_ monoliths with smooth skeletons and well-defined mesopores can be obtained, and the BET surface area is enhanced to 583.8 m^2^ · g^−1^.

## 1. Introduction

As an important structural and functional material, zirconia (ZrO_2_) has attracted considerable attention because of superior thermal, mechanical, chemical stability and electrical properties [1–3]. Zirconia has been extensively applied as an advanced material in various areas such as electronics, optics, catalysis and high-temperature structural engineering [4–10]. The tetragonal phase of ZrO_2_ has both acidic and basic properties and gives the most active catalyst for several catalytic reactions [11, 12]. Porous zirconia with precisely designed pore structures has become the new development of zirconia materials for catalyst supports, heat insulation, particle filters and gas membranes under severe conditions such as high-temperature and corrosive environments [13–18].

A lot of effort has been made to obtain porous zirconia such as pore-forming agents, tape-casting, gel casting, templating, impregnation and injection molding [19–23]. Sakka *et al* [24] reported a template-assisted method to fabricate macroporous ceramics consisting of TiO_2_ and ZrO_2_. The sol-gel method accompanied by phase separation is known as a promising technique for fabricating monolithic materials with a hierarchical porous structure [25, 26]. This method has been used to fabricate porous SiO_2_ [26], TiO_2_ [27–30] and Al_2_O_3_ [31]. In our previous works, we have demonstrated the preparation of monolithic macroporous mayenite [32], mullite [33], cordierite [34] and AlPO_4_ [35] by this approach. Konishi *et al* [36] reported the preparation of monolithic zirconia gels by this route. However, they used highly reactive and expensive zirconium propoxide (Zr(O^n^Pr)_4_) as the starting materials under a strongly acidic condition, and the process and the pore structure are too hard to control on account of the rapid reaction rate.

In this work, we demonstrate the novel and facile preparation of a porous zirconia monolith via the sol-gel process, accompanied by phase separation. The ionic precursors (zirconium oxychloride, ZrOCl_2_ · 8H_2_O) are utilized to synthesize a zirconia monolith in the presence of propylene oxide (PO) and poly(ethylene oxide) (PEO). The gelation of the system is mediated by PO, whereas PEO is added as a phase separation inducer. The resulting zirconia monoliths possess precisely controllable macropores at suitable starting compositions, and the solvothermal treatment of the resultant monoliths can further yield a high density of mesopores in crystallized skeletons, leading to a remarkable increase of surface area. The synthesis process has several advantages compared to the traditional alkoxide-derived sol-gel route, which allows the technique to involve various transition metals.

## 2. Experimental details

### 2.1. Materials

Zirconium oxychloride (ZrOCl_2_ · 8H_2_O) was used as a zirconium source. Mixtures of distilled water (H_2_O) and ethanol (EtOH, Sinopharm Chemical Reagent Co., Ltd, China, AR) were used as the solvents. PO (Sigma-Aldrich Co., USA, 99.5%) was used to initiate gelation, and PEO (Aladdin Co., China, AR) having an average molecular weight (*M*_*w*_) of 1 × 10^6^ was used as the polymer to induce phase separation. All chemicals were used as received.

### 2.2. Preparation of monoliths

The starting compositions were listed in table 1. In a typical synthesis, 1.610 g of ZrOCl_2_ · 8H_2_O and *W*_PEO_ g of PEO were dissolved in a mixture of *V*_H2O_ mL of distilled water and *V*_EtOH_ mL of ethanol. After continuously stirring for 120 min, *V*_PO_ mL of PO was then added to the transparent solution under ambient conditions (25 °C). After stirring for 1 min, the resultant homogeneous solution was transferred into a container. The container was sealed and kept at 60 or 80 °C for gelation. After gelation, the obtained wet gels were aged at 40 °C for 72 h and then evaporation-dried at 60 °C for 72 h. Some of the resultant xerogels were subsequently heat-treated at various temperatures up to 1000 °C for 2 h with a heating rate of 2–3 °C · min^−1^. Some of the wet gels were immersed in 0.5, 1.0 and 2.0 mol L^−1^ NH_4_OH solution at 40 °C three times, respectively. Then, the wet gels with solvent were together transferred into a stainless-steel autoclave vessel with a Teflon inner lining and additionally aged at 180 °C for 12 h under an autogeneous pressure in order to tailor the micro- and mesoporous structures. The solvothermally aged gels thus obtained were solvent-exchanged with distilled water and 2-propanol and were evaporation-dried at 40 °C for 5 d.

**Table 1.** Starting compositions of the samples.

Sample No.	ZrOCl_2_ · 8H_2_O g^−1^	H_2_O (*V*_H2O_) mL^−1^	EtOH (*V*_EtOH_) mL^−1^	PO (*V*_PO_) mL^−1^	PEO (*W*_PEO_) g^−1^
P1	1.610	2.0	2.4	0.50	0.100
P2	1.610	2.0	2.4	0.52	0.100
P3	1.610	2.0	2.4	0.54	0.100
P4	1.610	2.0	2.4	0.56	0.100
P5	1.610	2.4	2.4	0.52	0.060
P6	1.610	2.4	2.4	0.52	0.075
P7	1.610	2.4	2.4	0.52	0.090
P8	1.610	2.4	2.4	0.52	0.105
P9	1.610	2.4	2.4	0.52	0.120
P10	1.610	2.4	2.4	0.52	0.135
P11	1.610	1.8	3.0	0.52	0.115
P12	1.610	2.0	2.8	0.52	0.115
P13	1.610	2.2	2.6	0.52	0.115
P14	1.610	2.4	2.4	0.52	0.115
P15	1.610	2.6	2.2	0.52	0.115

### 2.3. Characterization of monoliths

Morphologies of the monolithic gels after drying, heat treatment and solvothermal treatment were observed by a field-emission scanning electron microscope (FESEM: SIRION-100, FEI Co., the Netherlands, with Au coating). Differential thermal analysis/thermogravimetry (DTA/TG; CRY-2P and WRT-3P, INESA, China) was performed at temperatures up to 1100 °C at a heating rate of 10 °C · min^−1^ while continuously supplying air at a rate of 100 mL · min^−1^. Chemical bonding in the dried gels was investigated with Fourier transform infrared spectroscopy (FTIR: Nicolet 5700, ThermoFisher Co., USA) using the potassium bromide (KBr) pellet technique. The crystal structure was confirmed by powder x-ray diffraction (XRD: XRD-6000, Shimadzu Corporation, Japan) using Cu *Kα* (*λ* = 0.154 nm) as an incident beam. Macropore size distributions over the diameter from 20 nm to 100 *μ*m were evaluated by mercury porosimetry (AutoPore IV 9510, Micromeritics Instruments, USA). Meso- and micropores were characterized by N_2_ adsorption−desorption isotherms (Autosorb-1-C, Quantachrome Instruments, USA). Before the N_2_ adsorption−desorption measurement, the samples were degassed at 100 °C under vacuum. The pore size distribution was calculated from them by the Barrett–Joyner–Halenda (BJH) method, and the surface area was obtained by the Brunauer–Emmett–Teller (BET) method. The bulk density of each sample was measured by mercury porosimetry. The porosity (%) of each sample was calculated as [(1 − *ρ*_*b*_)/*ρ*_*s*_] × 100, where *ρ*_*b*_ and *ρ*_*s*_ refer to the bulk and skeletal densities, respectively.

## 3. Results and discussion

### 3.1. Formation of macroporous monoliths

In this system, zirconium oxychloride is used as the ionic precursor, PEO is used as the phase separation inducer and PO is used to mediate the sol-gel process. The epoxide-mediated sol-gel reaction was firstly reported by Gash *et al* [37–39]. The mechanism can be illustrated as follows. (1) Epoxide added in the homogeneous solution is protonated by H^+^. (2) An irreversible ring-opening reaction is brought about by nucleophilic groups such as H_2_O, Cl^−^ and NO_3_^−^. The processes of this system are shown as the following reaction equations. The overall reaction can raise the pH of the solution quickly and uniformly, which promotes the hydrolysis and condensation.1
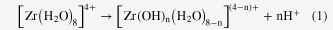


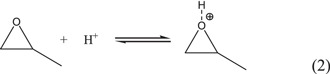

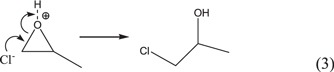

4
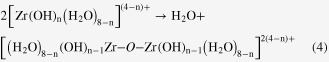


The phase separation tendency of the polymeric system can be evaluated by the Flory–Huggins theory [40–42]. The Gibbs free energy change of mixing, Δ*G*, can be described as follows:5
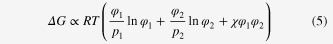

**Figure 1.**
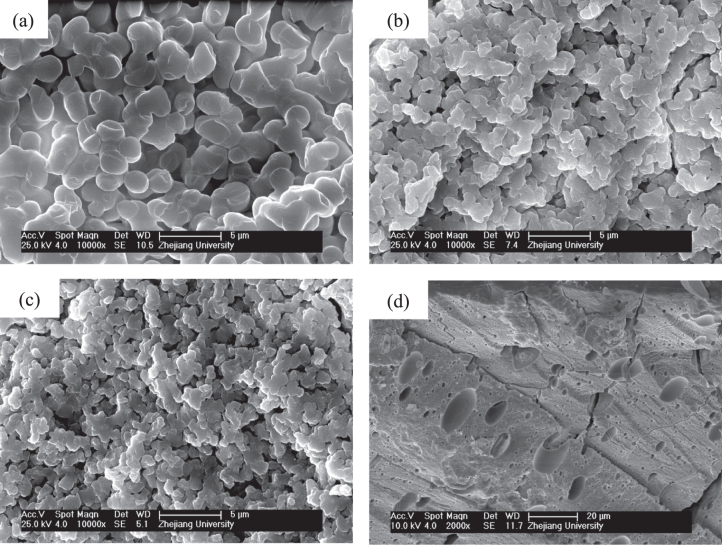
SEM images of dried ZrO_2_ gels prepared with various *V*_PO_: (a) 0.50 mL (P1), (b) 0.52 mL (P2), (c) 0.54 mL (P3) and (d) 0.56 mL (P4).

6
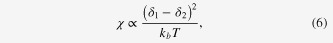
where *φ*_*i*_, *P*_*i*_ and *δ*_*i*_ are the volume fraction, degree of polymerization and solubility parameter of component *i* (*i* = 1 or 2), respectively. *k*_*b*_ is the Kauri–Butanol value, *R* is the gas constant and *T* is the absolute temperature. The former two terms in parentheses represent the entropic contribution, and the last term is the enthalpic contribution. The effects of the main starting compositions (PO and PEO contents) on the phase separation and macrostructure formation of ZrO_2_ dried gels will be investigated as follows:

The SEM microphotographs of the dried gels with different PO contents are shown in figure 1. It can be seen that the *V*_PO_ has a great impact on the macroporous structure of ZrO_2_ monoliths. The morphology of gels changes from fine aggregated particles to isolated macropores. When *V*_PO_ is small, the sol-gel process freezes the late stage morphology of phase separation; only aggregated particles are obtained (figure 1(a)). The increasing *V*_*PO*_ can dramatically shorten the gelation time of the system. Therefore, the early stage morphology of phase separation is acquired when too much PO is added (figure 1(d)).

**Figure 2.**
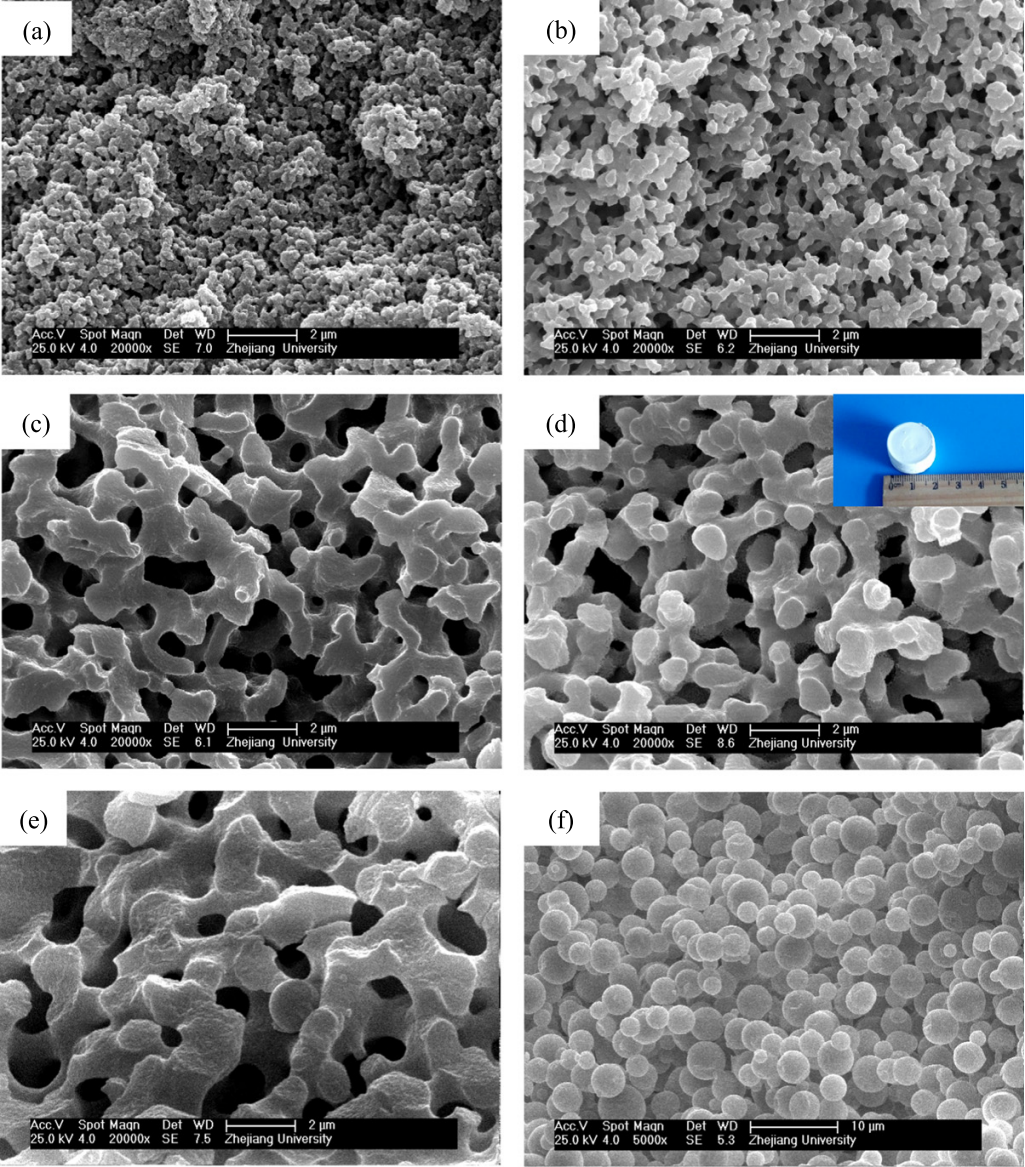
SEM images of dried ZrO_2_ gels prepared with various *W*_PEO_: (a) 0.060 g (P5), (b) 0.075 g (P6), (c) 0.090 g (P7), (d) 0.105 g (P8), (e) 0.120 g (P9) and (f) 0.135 g (P10); the insect picture in (d) is the appearance of the dried monolith of the P8 sample.

Figure 2 presents the morphologies of dried gels prepared with different *W*_PEO_. With the increasing *W*_PEO_, the morphology of gels changes from nanopores (figure 2(a)), through co-continuous skeletons and pores (figures 2(b)–(e)), to particles (figure 2(f)), indicating the rising phase separation tendency. An appropriate proportion of PO and PEO can lead to a concurrent sol-gel transition and phase separation, producing co-continuous porous structures. The system is divided into the co-continuous gels phase and the fluid phase. The fluid phase is transformed into macropores after evaporation drying. As a result, monolithic dried gels with interconnected macropores and co-continuous skeletons are obtained.

**Figure 3.**
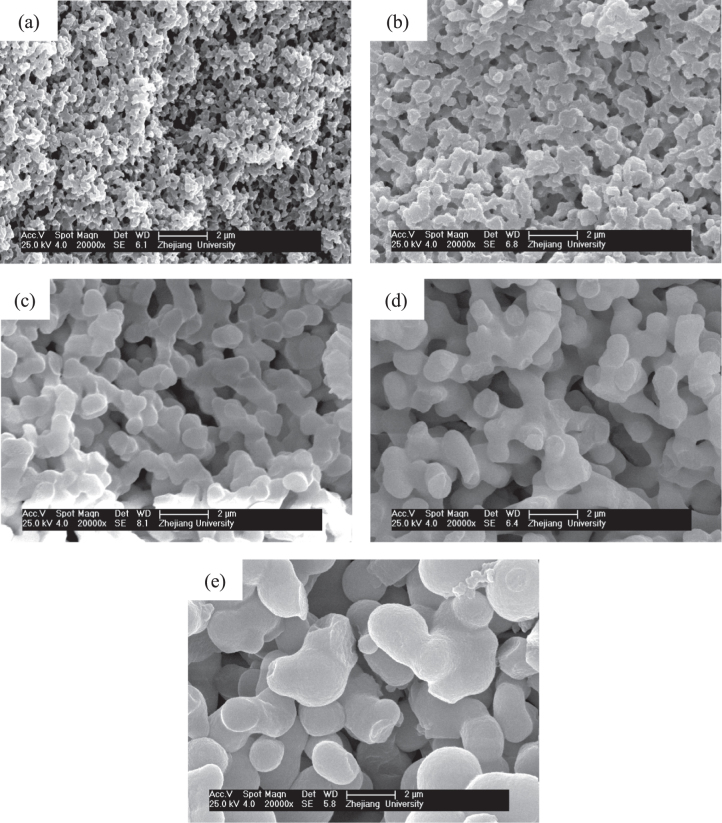
SEM images of dried ZrO_2_ gels prepared with various solvent ratios (*V*_H2O_/*V*_EtOH_): (a) 1.8/3.0 (P11), (b) 2.0/2.8 (P12), (c) 2.2/2.6 (P13), (d) 2.4/2.4 (P14) and (e) 2.6/2.2 (P15).

From the above equations (5) and (6), it can be seen that the increasing *χ*, which can be caused by the enlarging polarity difference of the gelation phase and mixed solvents, can also alter *ΔG* from negative to positive. As we have known, the polarity of the gel phase gradually decreases during the poly-condensation because of the consumption of high polarity hydroxy groups, and the polarity of the mixed solvents rises due to the enlarging proportion of water. We also investigate the influence of different solvent proportions on the morphology of dried gels, as presented in figure 3. The morphologies of the dried gels change from nanopores, through co-continuous skeletons, to broken bulky skeletons with the increase of water proportion. When the proportion of water in the mixed solvents is low, the polarity difference between the gel phase and mixed solvents becomes small and leads to a weak phase separation tendency (figure 3(a)). In contrast, the phase separation tendency in the system becomes stronger with a higher proportion of water, and the broken bulky skeletons are obtained (figure 3(e)).

**Figure 4.**
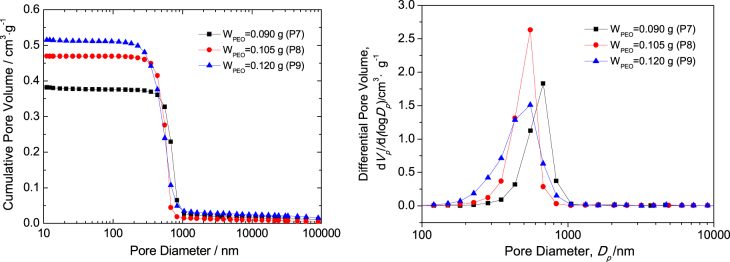
Macropore size distribution of the dried ZrO_2_ monolith with various PEO contents evaluated by mercury porosimetry.

The macropore size distributions of P7, P8 and P9 dried gels with interconnected macropores and co-continuous skeletons are shown in figure 4. Mercury porosimetry analysis indicates that the gels possess a narrow pore size distribution, reflecting the macrostructure formed via the spinodal decomposition [25]. The macropore size of the dried gels is distributed roughly between 0.3 and 1 μm, and the median macropore sizes of P7, P8 and P9 dried gels are 0.68, 0.55 and 0.55 *μ*m, respectively. The macropore size distribution of the sample with 0.105 g PEO (P8) is narrower than the two others, indicating ideal macroporous structure. The bulk densities of the three dried gels are 1.21, 1.08 and 1.01 g · cm^−3^, corresponding to 20.5%, 18.3% and 17.1% of the theoretical density (5.89 g · cm^−3^); the total pore volumes are 0.399, 0.473 and 0.524 cm^3^ · g^−1^; and the total porosities are 48.4%, 51.2% and 52.9%, respectively. It indicates that the pore size, pore volume and porosity increase, and the bulk density of dried gels decreases with the increase of PEO content. The results agree well with the SEM morphology.

**Figure 5.**
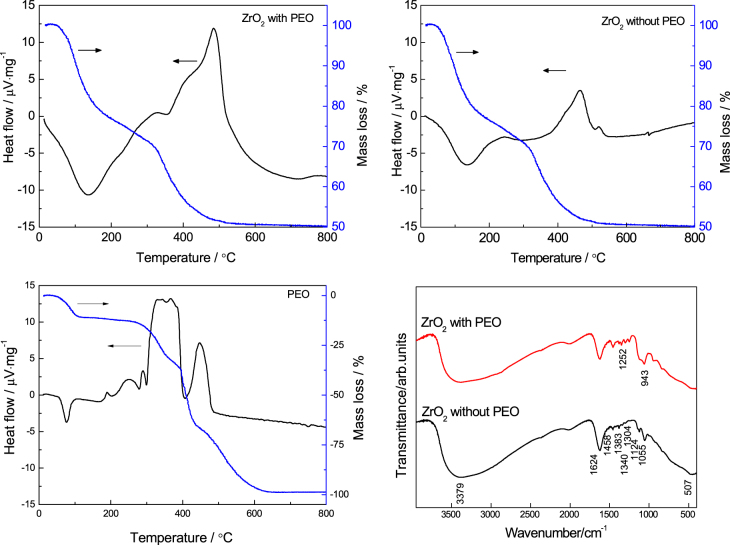
DTA/TG curves and FTIR spectra of dried ZrO_2_ gels with and without PEO.

To clarify the distribution of PEO between the gel phase and liquid phase, thermal and infrared analyses were carried out, as shown in figure 5. There is an exothermal peak that resulted from the decomposition of PEO between 300 and 400 °C in the gels prepared with PEO; this peak does not appear in the DTA curve of the gels prepared without PEO. In the FTIR spectra two new peaks appear around 1252 and 943 cm^−1^ in the gel prepared with PEO. They correspond to the asymmetric torsional vibration and to the rocking vibration of the CH_2_ group [43, 44], respectively. The peak around 1124 cm^−1^, which originates from stretching of the C–O–C bond [45], is stronger in the gel prepared with PEO than in the PEO-free gel. These results confirm the presence of PEO in the dried gel fabricated with PEO. They suggest that in this ZrO_2_ system, similar to the PEO-incorporated alkoxy-derived SiO_2_ sol-gel [26], PEO is absorbed on the surface of ZrO_2_ oligomers through hydrogen bonds [46–48], which can increase the hydrophobic–hydrophilic repulsive interaction with solvent mixtures and finally cause phase separation.

### 3.2. Heat treatment of porous monoliths

According to the DTA curves of the dried gels, the heat treatment of the dried gel (P8 sample) was carried out between 800 and 1100 °C for 8 h with a heating rate of 2–3 °C · min^−1^. The impact of heat treatment on the crystallization and porous structure is examined below.

**Figure 6.**
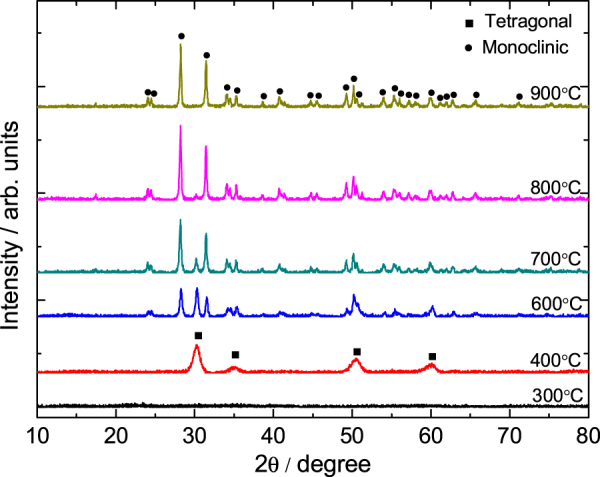
XRD patterns of a ZrO_2_ monolith heat-treated at various temperatures.

**Figure 7.**
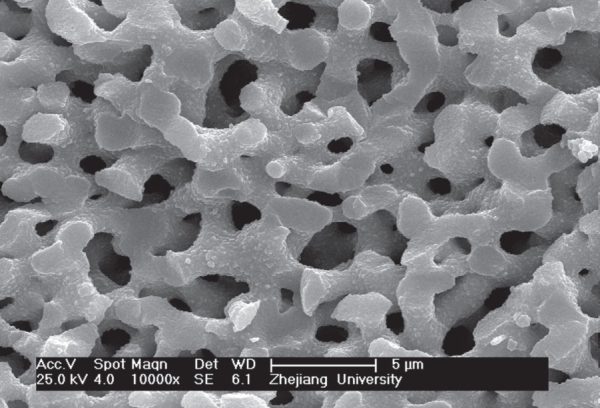
SEM image of ZrO_2_ monoliths after heat treatment at 700 °C

Figure 6 displays the XRD patterns of the gels heat-treated at various temperatures. No peaks are observed for the gels heat-treated at 300 °C, which manifests an amorphous state. After calcination at 400 °C, broad diffraction peaks appear due to the precipitation of tetragonal ZrO_2_, and all tetragonal ZrO_2_ turn to monoclinic ZrO_2_ after calcination at 900 °C. From the Scherrer’s equation the crystallite size of tetragonal ZrO_2_ obtained at 400 °C is 7.6 nm, and the monoclinic crystallite size acquired at 900 °C is 35.4 nm. This means that increasing the heat-treated temperature can enlarge the tetragonal ZrO_2_ crystallite and finally transform it into monoclinic ZrO_2_ crystallite, which is more stable at a low temperature. The result is consistent with Garvie’s theory [49], which believes that tetragonal ZrO_2_ can exist in low temperatures when the nanoparticle size is less than 30 nm. Figure 7 presents the SEM image of gels heat-treated at 700 °C. It can be seen that the well-defined macroporous morphology is basically retained, signifying that the heat treatment does not destroy the macropore structure of ZrO_2_ monoliths.

**Figure 8.**
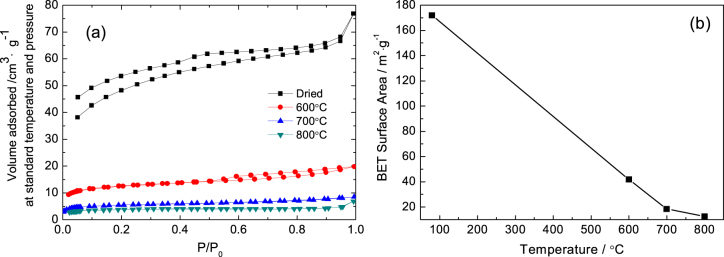
N_2_ adsorption–desorption isotherms (a), BET surface area (b) of ZrO_2_ monoliths heat-treated at various temperatures.

Figure 8 shows the N_2_ adsorption−desorption isotherms and BET surface area of gels heat-treated at various temperatures. The as-dried and 600 °C heat-treated gels exhibit isotherms of type-IV, while the gels heat-treated at 700 and 800 °C show isotherms of type-I (figure 8(a)). The results indicate that the elevating temperature of heat treatment can exterminate the mesopores of monoliths. It is also confirmed by the corresponding BET surface area (figure 8(b)). The BET surface area decreases from 172 to 13 m^2^·g^−1^ after being heat-treated at 800 °C due to the disappearance of mesopores, caused by phase transformation, and the aggregation of nanoparticles due to sintering.

### 3.3. Solvothermal treatment of porous monoliths

**Figure 9.**
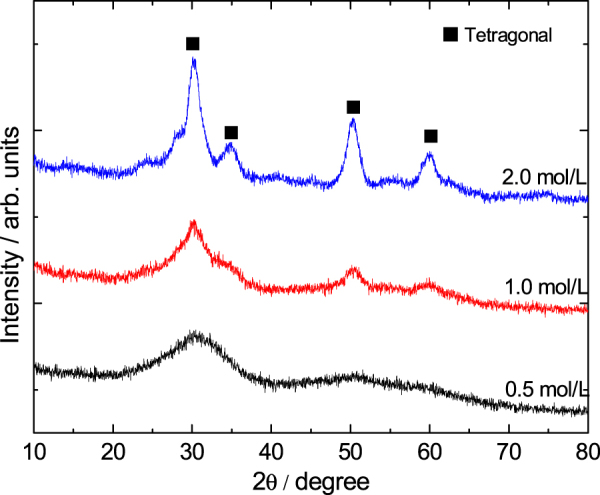
XRD patterns of ZrO_2_ monoliths after solvothermal treatment with various ammonia concentrations.

**Figure 10.**
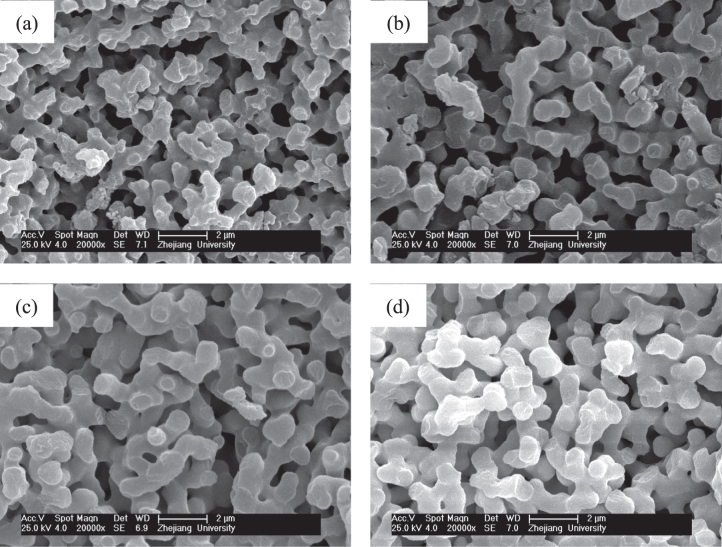
SEM images of ZrO_2_ monoliths before (a) and after solvothermal treatment with various ammonia concentrations: (b) 0.5 mol L^−1^, (c) 1.0 mol L^−1^ and (d) 2.0 mol L^−1^.

The solvothermal treatment of gels is introduced to study the effects on the crystallization and modification of ZrO_2_ skeletons. The choice of appropriate organic solvents plays a key role in the solvothermal synthesis, such as redox, polarity, complexation, viscosity, and so forth, and strongly influences the heterogeneous liquid–solid reactions [36]. In this study, an ethanol solution of ammonia was chosen as the solvent, and ZrO_2_ monoliths were solvothermally treated with various ammonia concentrations at 180 °C for 12 h. The XRD patterns shown in figure 9 demonstrates that the peaks of tetragonal ZrO_2_ crystallite become increasingly sharp when the ammonia concentration increases from 0.5 to 2.0 mol L^−1^. This is on account of the higher solubility of ZrO_2_ gel particles in the solvent with a higher concentration of ammonia and with the reaction of dissolution-precipitation, which will lead to the formation of the products with higher crystallinity at a rapid rate [26]. Figure 10 shows the macroporous morphology of ZrO_2_ monoliths before and after being solvothermally treated with different ammonia concentrations. It is observed that ammonia concentration does not much affect the tailoring on the skeletons of gels. The scale of the co-continuous skeleton slightly increases, and the surface of the skeletons just become smooth.

**Figure 11.**
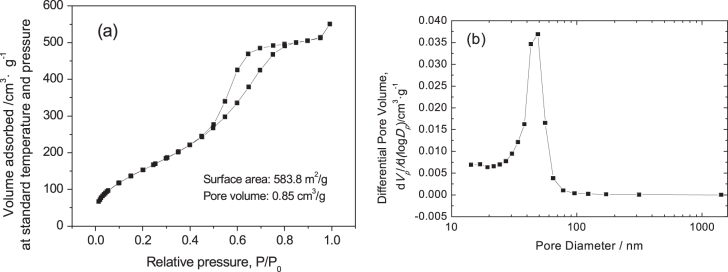
N_2_ adsorption–desorption isotherm (a) and BJH pore size distribution (b) of a ZrO_2_ monolith after solvothermal treatment with an ammonia concentration of 2.0 mol L^−1^.

During the drying stage of wet gels without solvothermal treatment, many micropores or/and mesopores in gel skeletons gradually decrease due to the capillary force, causing a large decrease in surface area. During solvothermal treatment of wet gels, the mesopores in the gel skeletons are regenerated via a process of dissolution/reprecipitation (Ostwald ripening). On the other hand, ammonia used as the solvent in the solvothermal treatment can improve the nucleation and growth of crystalline nanoparticles by providing more OH^-^ and can produce more mesopores constructed by nanoparticles. Moreover, the mesopores are not spoiled in the subsequent drying stage. The N_2_ adsorption–desorption isotherm of the ZrO_2_ monolith after solvothermal treatment exhibits isotherms of type-IV with an H1 hysteresis loop, indicating the existence of mesopores (figure 11(a)). Also, the BJH pore size distribution (figure 11(b)) also verifies that the pore size concentrates in the mesopore range. According to the calculation, the BET surface area of the monolith is as high as 583.8 m^2^· g^−1^, the average pore diameter is 58.3 nm and the average pore volume is 0.8508 cm^3^ · g^−1^. Compared to the as-dried gels, solvothermal treatment could generate a large amount of mesopores and significantly increase the average surface area and pore volume.

## 4. Conclusions

ZrO_2_ gels derived from a low-cost metal salt precursor have been synthesized by an epoxide-mediated sol-gel route, accompanied by phase separation. The appropriate choice of the amounts of epoxides, solvents and polymers allowed the formation of a gel with controlled macroporous morphology. Heat treatment of the dried gels at 400 and 600 °C results in the formation of tetragonal ZrO_2_ and monoclinic ZrO_2_, respectively, without spoiling the macrostructure. Solvothermal treatment with an ethanol solution of ammonia increases the number and size of the micropores and mesopores, thereby increasing the BET surface area from 171.9 to 583.8 m^2^· g^−1^. The presented synthesis process thus enables us to produce ZrO_2_ monoliths having bimodal meso-macroporous structures with adjustable pore sizes.

## References

[C1] Tanabe K (1985). J. Mater. Chem. Phys..

[C2] Mercera P, Vanommen J, Doesburg E, Burggaaf A, Ross J (1991). J. Appl. Catal..

[C3] Wirth H, Hearn M A (1995). J. Chromatogr..

[C4] Ortiz-Landeros J, Contreras-García M E, Pfeiffer H (2009). J. Porous Mater..

[C5] Wu C H, Chen S Y, Shen P (2013). J. Solid State Chem..

[C6] Jung W, Hertz J L, Tuller H L (2009). Acta Mater..

[C7] Gionco C, Paganini M C, Giamello E, Burgess R, DiValentin C, Pacchioni G (2014). J. Phys. Chem. Lett..

[C8] Larsen G, Lotero E, Petkovic L M, Shobe D S (1997). J. Catal..

[C9] Diaz-Torres L A, dela Rosa E, Salas P, Romero V H, Angeles-Chavez C (2008). J. Solid State Chem..

[C10] Fonseca F C, deFlorio D Z, Muccillo R (2009). Solid State Ion..

[C11] Yamaguchi T (1994). J. Catal. Today.

[C12] Centi G, Cerrato G, Dangelo S, Finardi U, Giamello E, Morterra C, Perathoner S (1996). J. Catal. Today.

[C13] Chuach G K (1999). J. Catal. Today.

[C14] Chuach G K, Jaenicke S (1996). J. Appl. Catal. A.

[C15] Minh N Q (1993). J. Am. Ceram. Soc..

[C16] Kim J D, Hana S, Kawagoe S, Sasaki K, Hata T (2001). J. Thin Solid Films.

[C17] Laurent M, Schreiner U, Langjahr P A (2001). J. Eur. Ceram. Soc..

[C18] Lee H L, Kim J T, Hong G G (1988). J. Korean Ceram. Soc..

[C19] Ortiz-Landeros J, Contreras-García M E, Gómez-Yáñez C, Pfeiffer H (2011). J. Solid State Chem..

[C20] Byrappa K, Adschiri T (2007). Prog. Cryst. Growth Char. Mater..

[C21] Liang X, Patel R L (2014). Ceram. Int..

[C22] Bluthardt C, Fink C, Flick K, Hagemeyer A, Schichter M, Volpe A (2008). Catal. Today.

[C23] Prete F, Ruzzuti A, Esposito L, Tucci A, Leonelli C (2011). J. Am. Ceram. Soc..

[C24] Sakka Y, Tang F Q, Fudouzi H, Uchikoshi T (2005). Sci. Technol. Adv. Mater..

[C25] Nakanishi K (1997). J. Porous Mater..

[C26] Nakanishi K, Soga N (1991). J. Am. Ceram. Soc..

[C27] Hasegawa G, Kanamori K, Nakanishi K, Hanada T (2010). J. Am. Ceram. Soc..

[C28] Konishi J, Fujita K, Nakanishi K, Hirao K (2006). Chem. Mater..

[C29] Konishi J, Fujita K, Nakanishi K, Hirao K (2004). Mater. Res. Soc. Symp. Proc..

[C30] Fujita K, Konishi J, Nakanishi K, Hirao K (2006). Sci. Technol. Adv. Mater..

[C31] Tokudome Y, Fujita K, Nakanishi K, Miura K, Hirao K (2007). Chem. Mater..

[C32] Guo X Z, Cai X B, Song J, Zhu Y, Nakanishi K, Kanamori K, Yang H (2014). New J. Chem..

[C33] Guo X Z, Li W Y, Nakanishi K, Kanamori K, Zhu Y, Yang H (2013). J. Eur. Ceram. Soc..

[C34] Guo X Z, Nakanishi K, Kanamori K, Zhu Y, Yang H (2014). J. Eur. Ceram. Soc..

[C35] Li W Y, Zhu Y, Guo X Z, Nakanishi K, Kanamori K, Yang H (2013). Sci. Technol. Adv. Mater..

[C36] Konishi J, Fujita K, Oiwa S, Nakanishi K, Hirao K (2008). J. Chem. Mater..

[C37] Gash A E, Tillotson T M, Satcher J H, Poco J F, Hmbesh L W, Simpson R L (2001). J. Chem. Mater..

[C38] Gash A E, Tillotson T M, Satchel J H, Hrubesh L W, Simpson R L (2001). J. Non-Cryst. Solids.

[C39] Gash A E, Satchel J H, Simpson R L (2004). J. Non-Cryst. Solids.

[C40] Huggins M L (1942). J. Am. Chem. Soc..

[C41] Huggins M L (1942). J. Phys. Chem..

[C42] Flory P J (1942). J. Chem. Phys..

[C43] McLachlan R D, Nyquist R A (1968). Spectrochim. Acta A: Molec. Spectrosc..

[C44] Durig J R, Zhen M, Heusel H L, Joseph P J, Groner P, Little T S (1985). J. Phys. Chem..

[C45] Liang C Y, Marchessault R H (1959). J. Polym. Sci..

[C46] Tokudome Y, Fujita K, Nakanishi K, Miura K, Hirao K (2007). Chem. Mater..

[C47] Saravanan L, Subramanian S (2005). J. Colloid Interface Sci..

[C48] Siffert B, Li J F (1989). J. Colloids Surf..

[C49] Garvie R (1978). J. Phys. Chem..

